# Pre‐Expanded Fronto‐Scalp Flaps Combined With Botulinum Toxin and Laser Therapy for Secondary Large‐Scale Craniofacial Defects

**DOI:** 10.1111/jocd.70624

**Published:** 2026-01-07

**Authors:** Feifei Chu, Shiqiang Liu, Yinke Tang, Jian Geng

**Affiliations:** ^1^ Department of Plastic Surgery, Xijing Hospital Fourth Military Medical University Xi'an China; ^2^ Department of Dermatology 987th Hospital of the Joint Logistic Support Force Baoji China

**Keywords:** botulinum toxin, fronto‐scalp flaps, secondary facial defects, tissue expander

## Abstract

**Background:**

Secondary facial defects remain a primary focus in plastic surgery, particularly large defects involving the midface, forehead, and scalp. While tissue expansion of the fronto‐scalp flap offers a viable reconstructive option, the lengthy expansion period and the issue of post‐expansion flap retraction necessitate further solutions.

**Objective:**

We conducted a prospective, comparative clinical study to investigate the role of Botulinum toxin in the expansion of fronto‐scalp flaps.

**Method:**

Between December 2019 and December 2024, 40 patients with large upper facial or scalp lesions were enrolled and randomly assigned to either the experimental group or the control group. Control group patients underwent tissue expansion combined with postoperative laser hair removal. Experimental group patients received the same tissue expansion and laser therapy, supplemented with Botulinum toxin injections administered both preoperatively (prior to expander implantation) and postoperatively in the forehead and scalp regions. We analyzed and compared parameters including expansion efficiency, post‐expansion flap retraction force, postoperative pain scores, and complication rates between the two groups.

**Results:**

Baseline characteristics showed no significant differences between the two groups before expander implantation. During the tissue expansion phase, the experimental group demonstrated significantly higher expansion efficiency compared to the control group. At both 3 and 6 months following flap transfer surgery, the post‐expansion flap retraction force was significantly lower in the experimental group than in the control group. However, no significant differences were observed in pain scores or complication rates between the two groups.

**Conclusion:**

Our prospective, comparative study demonstrates that Botulinum toxin application enhances the efficiency of tissue expansion using the fronto‐scalp flap for reconstructing large secondary facial and scalp defects. It also reduces post‐expansion flap retraction force, ultimately contributing to favorable functional and aesthetic surgical outcomes.

**Trail Registration:**

ChiCTR1900027702.

## 1 Introduction

As a vital anatomical structure and core identity marker, the face is essential to human function and social engagement [1]. Consequently, secondary facial defects have consistently been a primary focus and challenge in plastic and reconstructive surgery [2]. Etiologies leading to secondary facial defects include post‐traumatic scars, pigmented nevi, verrucous nevi, and facial tumors [3, 4, 5]. Multiple factors underlie the high incidence of these defects. Reconstructive options include skin grafting, local/regional flaps, and free flaps. However, each method entails limitations for large‐area facial defects. Although technically simpler, skin grafts are prone to postoperative contracture and pigmentation disorders [6]. Local non‐expanded flaps provide limited coverage. Free flaps, in contrast, involve greater surgical risk and technical complexity, often yielding bulky tissue [7]; thus, they remain a last‐resort option when conventional methods fail.

Tissue expanders offer distinct advantages for large facial defect reconstruction. Via gradual mechanical stretch, they stimulate adaptive tissue hyperplasia, generating sufficient tissue with ideal color, texture, and thickness for defect resurfacing [8, 9]. Essentially, tissue expansion represents a strategy of “trading time for tissue gain.” Large secondary facial defects predominantly involve the midface, forehead, and scalp. For these cases, the forehead‐scalp expanded flap represents an optimal reconstructive solution. Maturation of laser hair removal technology has streamlined this approach [10]. When distal flap perfusion is adequate, reliable reconstruction becomes achievable. Additional advantages include: (1) incision concealment within the hairline, and (2) enhanced aesthetic outcomes through postoperative laser ablation of hair follicles in transposed scalp tissue.

Despite optimized outcomes achievable with forehead‐scalp expanded flaps and postoperative laser hair removal for large secondary facial defects, significant clinical challenges persist. First, the protracted expansion period combined with peri‐expander pain—particularly prevalent in children with large pigmented nevi—frequently compromises treatment compliance [11]. This often precipitates expansion‐phase complications including displacement, exposure, and infection. Second, forehead muscle retraction following flap transfer can cause superior eyebrow displacement, impairing facial symmetry. Preclinical studies indicate Botulinum Toxin Type A (BTX‐A) reduces resistance in fasciocutaneous flap expansion, accelerates expansion rate, increases tissue gain, and decreases flap contraction [12]. Clinical evidence further suggests BTX‐A mitigates post‐expansion pain in breast reconstruction [13]. Therefore, building upon prior experience and related research, we designed a prospective controlled study to investigate whether BTX‐A can enhance the expansion efficiency of forehead‐scalp flaps, suppress postoperative retraction of the frontalis muscle and galea aponeurotica following expander removal, and reduce postoperative pain levels in patients.

## 2 Patients and Methods

This study, conducted in accordance with the Declaration of Helsinki with approval from the Ethics Committee of Xijing Hospital, obtained written informed consent from all participants or their legal guardians. Between December 2019 and December 2024, a total of 40 patients were enrolled. Inclusion criteria comprised: (1) presence of secondary facial defects in the midface, forehead, or scalp region; (2) large defect size necessitating tissue expansion for reconstruction; (3) age between 4 and 65 years; and (4) provision of signed informed consent. Exclusion criteria included: (1) comorbidities potentially affecting treatment; (2) history of prior Botulinum toxin injections or laser therapy in the facial region; and (3) withdrawal due to complications such as expander exposure or severe infection. Using a random number table method, the 40 patients were randomly assigned to two groups (*n* = 20 per group). The control group underwent standard tissue expander implantation followed by postoperative laser therapy. The experimental group received preoperative Botulinum toxin injection (prior to expander implantation) in addition to the same standard tissue expander implantation and postoperative laser therapy as the control group.

### 2.1 Surgical Technique

#### 2.1.1 Botulinum Toxin Injection

The experimental group received identical standard treatment plus two forehead injections of BOTOX (Allergan; ~40 units each) administered by a single surgeon—one 3 days pre‐expander implantation and one 3 days post‐flap transfer.

#### 2.1.2 Examination of Blood Vessels

All patients scheduled for axial pattern flap transfer in the second‐stage surgery underwent preoperative Doppler ultrasonography for vascular mapping 1 day prior to surgery. In the majority of cases within our cohort, the targeted vessel was the supratrochlear artery. This artery is typically located approximately 2 cm lateral to the facial midline and serves as the primary vascular supply for forehead flaps. In the mid‐forehead region, the supratrochlear artery enters the scalp near the hairline, coursing superiorly. Its branches anastomose with those of the supraorbital artery to collectively supply the scalp. For the minority of patients presenting with defects involving the lower eyelid or nasal region, the target vessel was the frontal branch of the superficial temporal artery (STA). The frontal branch of the STA typically branches 1–2 cm superior to the zygomatic arch; the frontal branch courses anteriorly and superiorly, while the parietal branch extends posteriorly and superiorly. Its surface projection can be marked along a line extending from 1.5 cm anterior to the tragus to a point 2 cm lateral to the lateral eyebrow.

#### 2.1.3 Implantation of Tissue Expander

**FIGURE 1:**
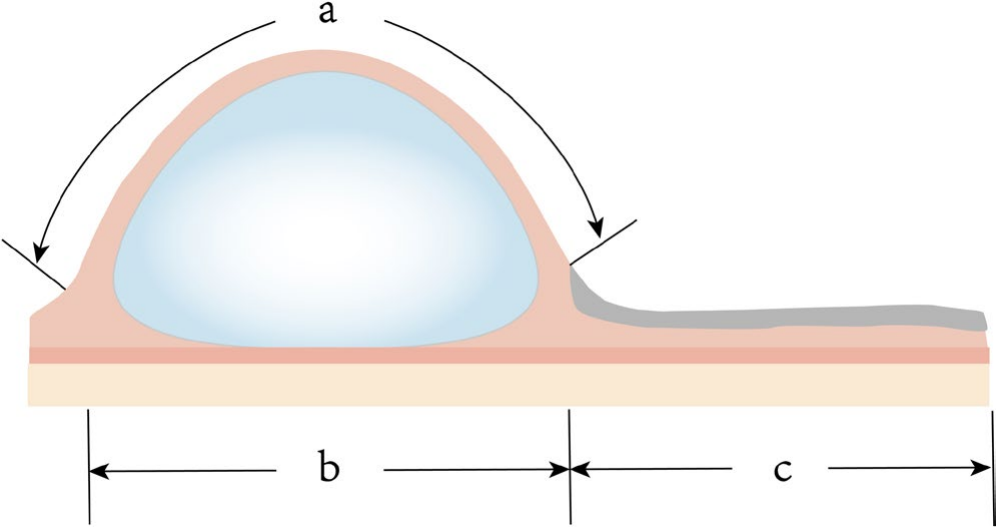
Criterion for determining adequate tissue expansion. The difference between the perimeter of the expanded flap (a) and the width of its base (b) approximates 1.1 times the width of the facial defect (c): a – b = 1.1c.

Following general anesthesia, expander size/shape was selected based on defect location and dimensions. For forehead/scalp defects, rectangular or cylindrical expanders (100–1000 mL) were implanted subgaleally over periosteum (forehead: subfrontalis plane to minimize bleeding). For nasal/lower eyelid defects, cylindrical expanders (100–800 mL) were placed beneath the superficial temporal fascia in the temporal region (exploiting its avascular, loose plane). A subcutaneous pocket ~1 cm larger than the expander was dissected. After inserting the fully flattened expander, a drain was placed and closure performed with interrupted non‐absorbable deep/full‐thickness sutures, followed by immediate intraoperative inflation (5–10 mL saline). Drains were removed when 24 h output was < 1 mL. Serial saline injections (10–30 mL/session, twice weekly, administered slowly) were adjusted by expander capacity and skin compliance, with elastic bandages positioned beneath the expander to prevent displacement. Expansion continued until meeting predetermined criteria: (1) Injected volume reached ~1.5× nominal expander capacity; (2) Difference between outer flap perimeter (a) and base width (b) approximated 1.1× defect width (c): a—b = 1.1c (Figure 1). Upon achieving these metrics, patients underwent expander removal and flap transfer.

#### 2.1.4 Flap Transfer

Following induction of general anesthesia supplemented by local anesthesia at the surgical site, the surgeon first excised the lesion. In cases where significant scar contracture was present, critical facial structures (e.g., eyes, nose) were restored to their anatomical positions after scar excision. The resulting wound was covered with moist gauze. The tissue expander was then completely removed and inspected for leakage or rupture. Based on the specific location and size of the defect, the surgeon designed an appropriate flap. If a local flap was selected, the defect was repaired using either transposition or advancement flap techniques. If a pedicled flap transfer was chosen, a delay procedure was performed at 3 weeks or flap division/inset was completed at 4 weeks post‐transfer. During subcutaneous suturing, sutures were spaced approximately 1.5–2 cm apart to optimize perfusion at the flap tip and prevent vascular compromise. Drains were placed, and the surgical site was dressed with a compression bandage. Postoperative dressing changes occurred every 2 days. Drains were removed when 24‐h output fell below 1 mL. Skin sutures on the face were removed at 7 days postoperatively, while scalp sutures were removed at 12 days.

#### 2.1.5 Laser Treatment

For patients presenting with residual hair follicles within non‐hair‐bearing facial areas following scalp flap transfer, laser hair removal was performed. Treatment commenced no earlier than 2–3 weeks post‐flap transfer surgery. An 800 nm diode laser system (LightSheer DUET) with a 9 mm × 9 mm spot size was utilized. Treatment parameters were incrementally escalated from low to high settings, with perifollicular papule formation serving as the clinical endpoint reaction. Based on individual patient needs, 3–6 treatment sessions were administered at 25–30 day intervals. Each session was followed by 30 min of localized ice application.

### 2.2 Assessment Item

The following parameters were evaluated across both groups: (1) Baseline characteristics including age, gender, defect etiology, lesion area, and expander capacity; (2) Expansion efficiency metrics (total injected volume and time required to achieve predetermined expansion criteria); (3) Postoperative assessments at 3 and 6 months, comprising: medial brow height change and pain intensity quantified using the Number Rating Scale [14] (NRS; 0–10 scale where 0 = no pain, 10 = worst imaginable pain); and (4) Incidence of postoperative complications.

### 2.3 Statistic Analysis

All statistical analyses were performed using SPSS 26.0 (IBM Statistics for Windows, IBM Corp., Armonk, NY, USA). Continuous variables are presented as mean or median as appropriate. Between‐group comparisons were analyzed using the Mann–Whitney U test, with *p* < 0.05 considered statistically significant.

## 3 Results

The mean age of patients in the experimental group was 20.8 years, comprising 10 males and 10 females; the mean age in the control group was 24.95 years, comprising 8 males and 12 females. Within the experimental group, there were 8 cases of pigmented nevus, 10 cases of scar, and 2 cases of verrucous nevus; in the control group, there were 7 cases of pigmented nevus, 10 cases of scar, 2 cases of verrucous nevus, and 1 case of benign tumor. The mean lesion area on the head and face was 166.60 cm^2^ in the experimental group and 167.16 cm^2^ in the control group; the mean expander volume was 399.00 mL in the experimental group and 385.22 mL in the control group. For all the above baseline characteristics, intergroup comparison showed no significant differences between the two patient groups (Table 1).

**TABLE 1:** Baseline data of patients.

	Experimental group	Control group	*p* value
Age, year	20.80 ± 8.83	24.95 ± 11.77	0.242
Gender	10 M, 10F	8 M, 12F	0.602
Etiology	Pigmented nevi 8 Scars 10 Verrucous nevi 2	Pigmented nevi 7 Scars 10 Verrucous nevi 2 Tumor 1	0.678
Lesion area, cm^2^	166.60 ± 76.04	167.16 ± 72.53	0.640
Expander capacity, mL	399.00 ± 170.51	385.22 ± 174.48	0.883

Regarding the expander inflation process prior to flap transfer (Table 2), the mean inflation volume was 596.25 mL in the experimental group and 614.25 mL in the control group, with no significant difference between groups. The mean expansion time was 112.40 days in the experimental group compared to 132.25 days in the control group (*p* = 0.035), indicating a significant difference. Postoperative flap retraction was analyzed by measuring changes in eyebrow height. At the 3‐month postoperative mark, the mean change in eyebrow height was 1.29 mm in the experimental group versus 3.56 mm in the control group (*p* < 0.001), showing a significant difference between groups. At 6 months postoperatively, the mean change was 1.38 mm in the experimental group and 3.94 mm in the control group (*p* < 0.001), again demonstrating a significant difference. For postoperative pain assessment using the NRS, the median score was 3 for both groups at 3 months postoperatively, with no significant difference. At 6 months postoperatively, the median NRS score was 2 for both groups, also with no significant difference (Table 3). Postoperative complications were also evaluated. In the experimental group, 1 patient developed a Stage I postoperative hematoma, and 1 patient experienced postoperative flap vascular compromise. In the control group, 3 patients exhibited expander exposure, displacement, and infection, and 1 patient developed postoperative flap vascular compromise. The occurrence of all complications showed no significant difference between the two groups (Table 4).

**TABLE 2:** Comparison of expansion rates between two groups.

	Experimental group	Control group	*p* value
Injected volume, mL	596.25 ± 259.95	614.25 ± 209.60	0.583
Expansion duration, d	112.40 ± 23.06	132.25 ± 23.15	0.035*

*Note:* *Statistical significance at *p* < 0.05.

**TABLE 3:** Comparison of flap contraction degree and NRS pain assessment.

	Experimental group	Control group	*p* value
Eyebrow height change, mm			
3 M postoperatively	1.29 ± 0.43	3.56 ± 0.84	0.00*
6 M postoperatively	1.38 ± 0.40	3.94 ± 0.72	0.00*
NRS pain assessment			
3 M postoperatively	3 (2.25, 4)	3 (2, 3.5)	0.298
6 M postoperatively	2 (1, 2)	2 (1, 2)	0.678

*Note:* *Statistical significance at *p* < 0.05.

**TABLE 4:** Complications postoperatively.

	Experimental group	Control group
Expander exposure	0	1
Expander displacement	0	1
Infection	0	1
Stage I hematoma	1	0
Flap vascular compromise	1	1

## 4 Cases Report

### 4.1 Case 1

**FIGURE 2:**
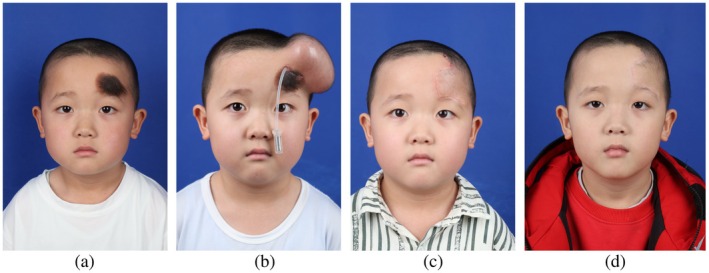
Postoperative comparison in a control group patient demonstrating discernible flap retraction at 6 months versus 10 days post‐surgery. (a) Preoperative presentation. (b) Status post tissue expander implantation. (c) Postoperative day 10 following flap transfer. (d) Postoperative month 6.

This male patient in the control group, aged 4 years, presented to our hospital with a large pigmented nevus on the forehead, measuring 5 cm × 3 cm. During stage I surgery, an 80 mL tissue expander was implanted in the forehead region. After 45 days of expansion, a total of 102 mL had been injected. The patient subsequently returned to our hospital for stage II flap transfer surgery. Considering the potential for postoperative flap retraction, advancement of the expanded flap was performed intraoperatively. Postoperatively, the flap demonstrated adequate blood perfusion. At 3 weeks post‐surgery, laser hair removal was performed on the flap, with a satisfactory outcome. Approximately 3 months postoperatively, retraction of the expanded flap was observed, resulting in an elevation of the eyebrow by 3.91 mm (Figure 2).

### 4.2 Case 2

**FIGURE 3:**
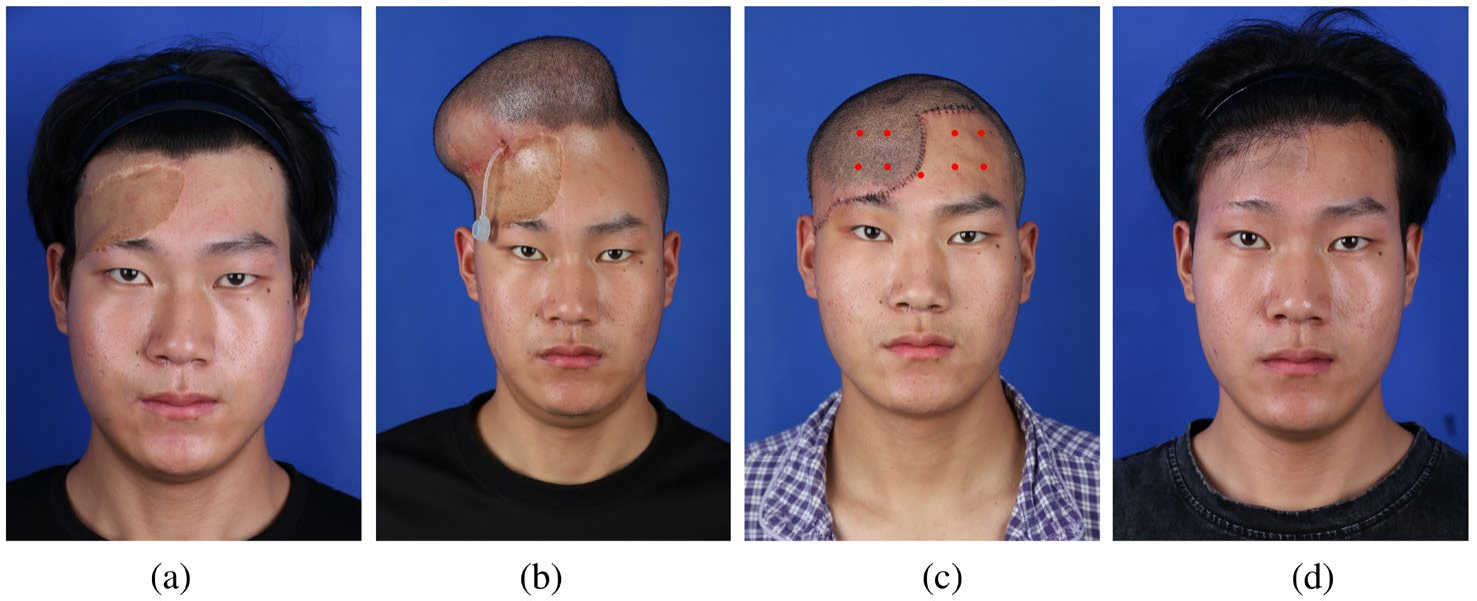
Serial photographs of an experimental group patient demonstrating minimal flap retraction and preserved eyebrow position. (a) Preoperative view. (b) Status post tissue expander implantation. (c) Postoperative day 3 following flap transfer (red dots indicate botulinum toxin injection sites). (d) Postoperative month 6.

This male patient in the experimental group, aged 22 years, presented to our hospital with a large scar on the right forehead, measuring 11 cm × 5.5 cm. Prior to expander implantation, 40 units of botulinum toxin were injected into both the forehead and scalp. Subsequently, a 400 mL tissue expander was implanted in the forehead region. After 115 days of expansion, a total of 617 mL had been injected. The patient then returned for stage II flap transfer surgery. Postoperatively, the flap demonstrated adequate blood perfusion. On postoperative day 3, two additional injections of botulinum toxin were administered, with a total dose of 40 units. At 3 weeks postoperatively, laser hair removal was performed on the flap, yielding satisfactory results. Approximately 3 months postoperatively, no significant retraction of the expanded flap was observed. Eyebrow height remained stable, with an elevation of only 0.86 mm (Figure 3).

## 5 Discussion

Tissue expansion was first introduced by Neumann in 1957 and began to be widely adopted clinically in 1976 [15]. Due to its ability to achieve favorable texture and color matching, this technique has gained broad application in the field of reconstructive surgery. Historically, the forehead flap was not considered an ideal choice for reconstructing large secondary defects in the mid‐upper face or scalp due to its broad pedicle base and high rate of donor‐site complications. Subsequently, with the refinement of various flap techniques and tissue expansion, the issues of significant donor‐site morbidity and excessive flap thickness associated with the forehead flap were substantially mitigated. Furthermore, when addressing large defects, two methods can be employed clinically to extend the forehead flap: one involves laterally turning the flap along the hairline, while the other incorporates scalp tissue into the flap design. In the past, prior to the advancement of laser hair removal technology, the preferred approach for managing large defects was lateral turning along the hairline to extend the forehead flap. However, because the supratrochlear artery courses vertically through the forehead region, this lateral turning design deviates from the main vascular axis. Consequently, the distal portion of a paramedian forehead flap may suffer from inadequate blood supply. Once insufficient perfusion occurs, necrosis of the distal flap segment becomes almost inevitable. Today, with the assistance of advanced laser hair removal technology, flap design has become considerably simpler. Provided the distal flap maintains sufficient blood perfusion, reconstruction of large mid‐upper facial and scalp defects has become significantly more reliable [16].

Previous animal studies by relevant scholars have demonstrated that botulinum toxin can eliminate isometric muscle contractions, thereby reducing resistance, decreasing myocutaneous flap contraction, and accelerating its expansion rate [12]. These findings provided valuable insights for our clinical approach. In our previous experience with forehead flap expansion, we primarily faced two key challenges. Firstly, a significant proportion of treated patients were children, a population presenting inherent clinical management challenges. Studies have shown that pediatric patients are more prone to expander‐related complications and exhibit relatively lower treatment compliance [11]. Prolonged expansion cycles further increase the risk of complications. Secondly, postoperative flap retraction has been a persistent problem. This retraction can lead to passive elevation of the eyebrow position, adversely affecting the final aesthetic outcome of the surgery. The mechanism of action demonstrated by botulinum toxin in animal studies directly addresses these clinical dilemmas. Our clinical research further validates the application value of botulinum toxin in forehead flap expansion, confirming its ability to enhance flap expansion efficiency and effectively reduce flap retraction force. Furthermore, current clinical research on the combination of tissue expansion and botulinum toxin is predominantly focused on the field of breast reconstruction, with related studies mainly concentrating on the analgesic effects of botulinum toxin [17]. However, findings across studies show some inconsistency. While some studies suggest botulinum toxin significantly alleviates postoperative pain, others express reservations about its analgesic efficacy. In our study, postoperative pain was quantitatively assessed using standardized scales. The results revealed no significant difference in pain scores between the experimental and control groups. This lack of difference may be attributed to the inherently marginal postoperative pain associated with conventional forehead flap expansion itself. Consequently, patients in the experimental group receiving botulinum toxin injections did not perceive a significant analgesic effect.

Our study also has certain limitations. First, the sample size was relatively small. Increasing the number of enrolled patients might have allowed differences in postoperative complication rates to reach statistical significance. Second, the follow‐up period was limited to 6 months postoperatively. Extending follow‐up to 1 year or longer would likely yield more conclusive results.

## 6 Conclusion

Our prospective comparative study demonstrates that botulinum toxin significantly enhances tissue expansion efficiency and mitigates postoperative flap retraction in expanded forehead‐scalp flap reconstruction of large secondary head and facial defects, thereby achieving favorable functional and aesthetic outcomes.

## Author Contributions

F.C. and S.L. performed the research. Y.T. and J.G. supervised the research study. S.L. analyzed the data. F.C. and S.L. wrote the paper.

## Funding

The authors have nothing to report.

## Ethics Statement

All procedures performed in studies involving human participants were in accordance with the ethical standards of the institutional and/or national research committee and with the 1964 Helsinki declaration and its later amendments or comparable ethical standards.

## Conflicts of Interest

The authors declare no conflicts of interest.

## Data Availability

The data that support the findings of this study are available from the corresponding author upon reasonable request.
